# Pyrolytic formation and photoactivity of reactive oxygen species in a SiO _2_/carbon nanocomposite from kraft lignin

**DOI:** 10.12688/f1000research.16080.1

**Published:** 2018-09-28

**Authors:** Dhanalakshmi Vadivel, Ilanchelian Malaichamy

**Affiliations:** 1 Department of Chemistry, Bharathiar University, Coimbatore, 641046, India

**Keywords:** ROS, photochemistry, methylene blue, degradation, UV

## Abstract

SiO _2_ and carbon produced by kraft lignin pyrolyzed at 600°C can generate stable reactive oxygen species (ROS) by reaction with atmospheric oxygen. In this study, we systematically investigate the photochemistry of peroxyl radicals in carbon-supported silica (PCS) and assess its effects on the methylene blue (MB) photodegradation. Characterization revealed that the higher ROS generation ability of SiO _2_/carbon under UV light irradiation was attributed to its abundant photoactive surface-oxygenated functional groups.

## Introduction

Consistent access to clean water has come into focus this millennium due to high pollution; a reduced amount of drinkable water could be the next challenge for the future due to overpopulation ^1– 3^. The application of photocatalytic technology using semiconductors to solve the environmental problems, like the degradation of organic effluents have been received much attention ^4– 8^. Heterogeneous photocatalysis using semiconductors is an interesting method falling into advance oxidation processes (AOPs) ^9– 11^ that can produce highly reactive species containing oxygen (ROS). In fact, with this method is possible to produce oxidizing molecules like hydrogen peroxide and singlet oxygen ( ^1^O _2_) together with radicals like hydroxyl radical (OH ^.^) and superoxide radical anion ( O _2_^.- ^) ^12– 13^. These reactants can decompose organic pollutants in wastewater giving harmless compounds ^14^.

Recently, N. Chen *et al*. reported that reactive oxygen species generation in hydrochar and photochemistry of Sulfadimidine degradation in water ^15^. Y. Chen *et al*. reported the photo degradation of tetracycline in aqueous solution under simulated sunlight irradiation through the singlet oxygen ^16^. Li *et al*. reported that the degradation of ibuprofen by UV–visible light irradiation included direct photolysis and self-sensitization via ROS ^17^. Wang *et al*. reported that when a simpler molecule without visible-light absorption is degraded, the Fe-hydroxyl complexes still promote the generation of ROS and thus accelerate degradation, although the pathway of electron transfer, and the mechanism of photocatalysis was not completely understood ^18^.

In literature are present many methods for photoassisted AOPs like photo-electrochemical cells composed by an anode made with boron-doped diamond and cathode in carbon nanotubes; with this system, a model azo dye was depleted ^19^. Also exfoliated graphene, decorated with titanium dioxide and nanoparticles, is effective for photo-catalytic water treatment ^20, 21^.

In our current scenario, stable peroxyl radicals in carbon-supported silica (PCS) are prepared from cheap starting materials. The method used is the pyrolysis under vacuum of kraft lignin deposited onto silica. Vacuum pyrolysis produced defective carbon bearing carbon radicals. These radicals are quickly transformed into peroxyl radicals by reaction with oxygen molecules present in the atmosphere.

## Methods

The materials and methods to produce PCS using high-vacuum pyrolysis are clearly explained and characterized previously ^22^. In brief, kraft lignin was absorbed onto silica and pyrolyzed under vacuum at 600 °C. For the kinetic data analysis, linear quadratic fitting and other kinetic fitting (reaction order checking) were performed by using Origin v6.0.

### Degradation of MB dye procedures and analyses

100-ml of air-equilibrated 10 ^-6^ M solutions of MB (Sigma Aldrich, India) in water containing 100 mg (1 mg/ml) of neat SiO _2_ or PCS were poured in quartz cylindrical reactors (90 mm diameter x 25 mm height). Solutions were magnetically stirred in the dark for 10 min before irradiation and kept under stirring during the experiment. The light source consisted of two 15-W phosphor-coated lamps (center of emission, 366 nm). Aliquots (4 ml) were withdrawn at 5-min intervals (for a total of 10-12 samples) during the irradiation until the disappearance of the color. Solids were removed by syringe filtration with a 0.4-µm pore size, and the filtrates immediately examined by UV-visible absorption spectroscopy in 1-cm quartz cuvettes using a JASCO V-630 UV-visible spectrophotometer. The absorbance was normalized by dividing the absorbance at 668 nm of the sample (A) with the absorbance of the initial solution (A _0_).

## Results and discussion

### Degradation of MB

**Figure 1.**
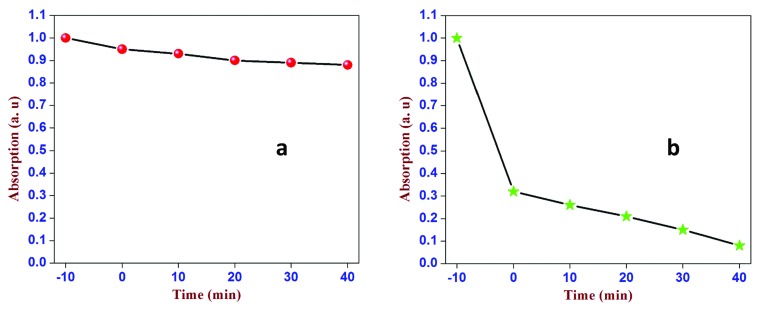
Normalized spectral intensity of the 668 nm band of methylene blue (MB) during ( **a**) the UV-irradiation of the MB/SiO _2_ suspension at 366 nm at different time intervals, and ( **b**) the same process for the MB/peroxyl radicals in carbon-supported silica (PCS) suspensions under otherwise identical conditions. The region between −10 and 0 min refers to the extent of adsorption of the MB dye under dark conditions. It shows the first-order kinetics of the photodegradation of the MB dye by MB/PCS. 3 repeats performed.

To assess the respective photocatalytic activity of PCS and of neat SiO _2_, we carried out competitive experiments with MB ( Figure 1). PCS did not react with MB, in fact, solutions left for 24 hours in the dark does not show a decrease of MB concentration. Nonetheless, under dark conditions the dye was absorbed by PCS to a nearly tenfold greater extent than with pristine SiO _2_ (dark region between −10 and 0 min, Figure 1b).

Normally photocatalysts produce radicals able to degrade organics but in the case of PCS the catalyst already possesses reactive radicals.

### Simple mechanism of established photocatalysts in MB

The net effect of PCS on the photodegradation of MB is a threefold increase in the kinetics of photodegradation ( Table 1). Without the assistance of an active photocatalyst, the only reaction mechanism that is applicable is the generation of singlet oxygen by sensitization ( Equation 2) via the excited state of the dye. The singlet oxygen can react with MB, giving rise to photobleaching ( Equation 3).

**Table 1.** Extent of adsorption and first-order kinetics of photodegradation of methylene blue (MB) (1.0 μM) on pristine SiO _2_ and on SiO _2_/graphene in aqueous media under ambient atmospheric conditions and under UV irradiation at 366 nm.

Dye	*k* (min ^−1^)		Adsorption, %	
	SiO _2_	PCS	SiO _2_	PCS
MB	0.027 ± 0.005	0.092 ± 0.006	24	91

Dye + photon = Dye*                                   (1)

Dye* + O _2_^T^ =Dye + O _2_^s^                             (2)

O _2_^S^ + Dye = oxidation products                  (3)

With PCS, MB is strongly absorbed onto the pyrolytic carbon present on the catalyst surface. Moreover, pyrolytic carbon possesses a high concentration of peroxyl radicals. The enhancement on the reaction kinetic could be due to a local increase of concentration of dye and active oxygen. Since the oxygen is reversibly absorbed on the carbon giving peroxyl radicals ^22^, the surface of the catalyst is never depleted due to the presence of oxygen in solution.

In fact, in these conditions, we can have, together with Equation 1– Equation 3, a possible reaction of the excited state of the reactant with peroxyl radicals or adsorbed oxygen on PCS ( Equation 4).

Dye* + PCS-OO = PCS + dye oxidation      (4)

The peroxyl radicals are reversibly formed by capture of atmospheric oxygen due to the presence of highly active pyrolytic carbon on PCS:

PCS + O _2_ = PCS-OO                                  (5)

Another possibility is the transfer of energy (or sensitization) of the excited state of the absorbed dye directly to the defective pyrolytic carbon, giving rise to formation of ROS. All these mechanism lead to an enhancement on the degradation of MB.

Raw data for the article ‘Pyrolytic formation and photoactivity of reactive oxygen species in a SiO2/carbon nanocomposite from kraft lignin’ are presented Click here for additional data file. Copyright: © 2018 Vadivel D and Malaichamy I 2018 Data associated with the article are available under the terms of the Creative Commons Zero "No rights reserved" data waiver (CC0 1.0 Public domain dedication).

## Conclusion

This study has shown that silica can be coated successfully with pyrolytic carbon obtained from inexpensive waste materials, such as kraft lignin and silica. The pyrolytic process performed at 600°C did not affect the crystalline state of silica when it was coated with carbon. The photocatalytic activity was measured against pristine SiO _2_ through an examination of the kinetics of degradation of MB by UV-vis spectroscopy. Under UV light irradiation, the degradation was threefold greater for the MB-PCS compared with MB-silica.

## Data availability

The data referenced by this article are under copyright with the following copyright statement: Copyright: © 2018 Vadivel D and Malaichamy I

Data associated with the article are available under the terms of the Creative Commons Zero "No rights reserved" data waiver (CC0 1.0 Public domain dedication). 

Dataset 1: Raw data for the article ‘Pyrolytic formation and photoactivity of reactive oxygen species in a SiO2/carbon nanocomposite from kraft lignin’ are presented, 10.5256/f1000research.16080.d218907 ^23^
