# Supplementary material for: Pyrolytic formation and photoactivity of reactive oxygen species in a SiO 2/carbon nanocomposite from kraft lignin
Source: F1000Res. 2018 Sep 28;7:1574. [Version 1] doi: 10.12688/f1000research.16080.1 (PMC6464069; doi:10.12688/f1000research.16080.1)
Supplement: Peer review history

# Peer review: Reviewer response for version 1

**DOI:** 10.5256/f1000research.17560.r46596

**Published:** 2019-04-11

**Authors:** Stefano Crespi

**Affiliations:** 1 Institute for Organic Chemistry, University of Regensburg, Regensburg, Germany

Vadivel and Malaichamy report on the activity of stable peroxyl radical species generated on carbon-supported silica after pyrolysis of kraft lignin deposited onto SiO _2_.

 The synthetic method applied is crucial to generating carbon radicals on the surface of the catalyst. These species can react with oxygen, readily forming stable ROS on the catalyst itself.

 The photocatalytic activity of these peroxidic reactants is tested against the photobleaching of methylene blue, providing a complete analysis of the results obtained.

 The article herein presented has a structured scholar presentation that is based on the authors’ previous work. The literature cited is coherent and adequate with the topic.

 As minor comments, the authors should pay attention to the citation of some of the references that got mixed up in the version presented, e.g. Y. Chen in the text (ref 16) is stated correctly, however it is wrong in the reference section, where names and surnames are cited incorrectly.

 To improve the readability, please write “Methylene Blue” in its full extent along with “MB” the first time it appears in the main body of the article, because the extended name is present only in the abstract.

 The study is based on the experience and methodology that the authors have recently published on Kraft lignin and its pyrolysis. Being experts in the field, they have devised a carefully planned work in all its aspects, comprising the synthetic, photochemical and analytical part.

 The authors provide the reader with a schematic, yet very precise method section. It is highly appreciated the author’s attention in giving detailed information on the specifics of all the instruments used, e.g. explicitly reporting the wavelength used for the irradiation (366 nm) when in several reports only generic data is given to the reader.

 All the data are repeated and checked three times. The average and the standard deviation is furnished in the text. The statistical interpretation of the data is adequate to the problem treated.

 The very low deviation found in the measures testifies the reproducibility of the method, which is remarkable, given the complex matrix analysed. All the data are accurately reported in a spreadsheet furnished as supplementary file to the article.

 The conclusions of the work are drawn in a schematic yet elegant way, summing up a nice work that is fully supported by the experimental evidence.

I have read this submission. I believe that I have an appropriate level of expertise to confirm that it is of an acceptable scientific standard.

# Peer review: Reviewer response for version 1

**DOI:** 10.5256/f1000research.17560.r38855

**Published:** 2018-10-05

**Authors:** Simone Lazzaroni

**Affiliations:** 1 Department of General Chemistry, University of Pavia, Pavia, Italy

This manuscript is focused on the application of supported stable peroxyl radicals for the photo-degradation of organic materials. In this work the authors systematically investigated the photochemistry of peroxyl radicals in carbon-supported silica (PCS) and then they evaluated the effects of PCS on the methylene blue photodegradation as a model for a generic organic effluent. The manuscript is clearly written with few errors (e.g. “N. Chen *et al.*” and “Y. Chen *et al.*” refer to the same reference article). However, the authors have extracted some interesting data that well supports the discussion and the appropriate conclusions. Furthermore, problems such as overpopulation and the lack of drinking water are unfortunately a plague that afflicts our entire planet. I encourage the authors to continue with their research, thus contributing to increase the impact of their study.

I have read this submission. I believe that I have an appropriate level of expertise to confirm that it is of an acceptable scientific standard.
